# Sulphonal

**Published:** 1889-11-23

**Authors:** 


### THERAPEUTICS.

### SULPHONAL.

We have heard a good deal latterly about sulphonal, to which chemists have given the synonym *diethyl-sulphon-dimethyl-methane*. It is produced by oxidation of a mixture of ethyl-mercaptan and acetone, and appears in the shape of white tabular crystals, tasteless and odourless. Its action is soporific, and it has been said not to affect digestion, pulse, or temperature. It has been regarded as especially efficacious in the sleeplessness of nervous patients. As usual with new preparations, it has probably been lauded more than it really deserves. Dr. D. J. Leech, physician to the Manchester Infirmary, says of sulphonal that its tastelessness has rendered it a favourite remedy, but that small doses such as 10 to 15 grains often fail, and that it may be necessary to raise the dose to 20 or 30 grains. Some have recommended 40 to 60 grains; but from doses above 20 grains Dr. Leech thinks troubles affecting the nervous system are sufficiently common to render warning to the patient of their possible occurrence desirable. In the discussion which took place at the Leeds meeting of the British Medical Association in August last, on recently introduced hypnotics and analgesics, Dr. Cranstoun Charles, lecturer on practical physiology at St. Thomas's, stated he had used sulphonal with the best results. The sleep was produced slowly, but it was light and refreshing, and no deleterious effects were produced on the circulation. In one case only had he observed any serious after effects, when headache, hysterical attacks, some hyperæsthesia, with thickness of speech, giddiness, and temporary loss of power in the muscles of the legs occurred to a nervous girl aged sixteen. A recent number of the *Philadelphia Therapeutic Gazette* contains an article on "The Unpleasant Effects of Sulphonal," by Dr. T. P. Crozer Griffiths, instructor in clinical medicine in the University of Pennsylvania. First to be noticed among the disadvantages of sulphonal is its slowness of action in producing sleep. This must be carefully borne in mind in determining the hour of its administration, and the patient should be notified of the peculiarity. Kast, who first employed the drug, advises it should be administered in a finely-pulverised condition in a warm fluid, with the evening meal, about seven or eight o'clock. Another disadvantage is the marked tendency which the hypnotic action shows to persist during the succeeding day. This persistence of hypnotic action has been frequently noticed (*vide British Medical Journal*, May 26, 1888). For this action of sulphonal no remedy is proposed except careful adaptation of the quantity to the individual. But this can only be ascertained by experience. Dr. Griffith writes: "I have frequently found that the amount necessary to produce satisfactory sleep was followed by the prolonged effects on the following day, or by various unpleasant after effects, while any smaller amount failed to bring about the desired effect during the night." On the other hand, a small dose merely served to render the patient uncomfortable during the night, and upon the following day. The unpleasant effects noticed by Dr. Griffiths are restless sleep and tendency to random talk during sleep; in one case, sleep-walking; afterwards a sense of depression, feeling of fatigue, and confusion of mind; in two instances there was a disturbance of gait; erythematous eruption over the breasts, and a cyanosed and semi-conscious state have also been noted by other authors. These symptoms may appear after large or quite small doses. Finally, it very often fails to exert any hypnotic effect in any dose. Altogether, the record of sulphonal is not very satisfactory. When it is found from experience that the action of a drug not only varies greatly with different persons, but also at different times with the same individual, much confidence cannot be placed in it. It may be said that such is the case with other hypnotics, and this is true to a certain extent, for there are peculiar idiosyncrasies for which we cannot account. But with other hypnotics the rule is, that the action on the same person at different periods does not vary much. Dr. Griffiths states that he has a decided preference for *paraldehyde* and especially for *amyleni hydrate.* With the latter drug his experience has been especially satisfactory, and disagreeable results following its administration are less frequently reported than in the case of sulphonal. Paraldehyde is contained in, and is probable the principal therapeutic agent, in the old *spiritus ætheris nitrosi* of the British Pharmacopœia. Amyleni hydrate has not been used so much in this country as in America, where it is regarded as a reliable hypnotic by various practitioners.

